# Bony cranial ornamentation linked to rapid evolution of gigantic theropod dinosaurs

**DOI:** 10.1038/ncomms12931

**Published:** 2016-09-27

**Authors:** Terry A. Gates, Chris Organ, Lindsay E. Zanno

**Affiliations:** 1 Department of Biological Sciences, North Carolina State University, Raleigh, North Carolina 27695, USA; 2 Paleontology Research Laboratory, North Carolina Museum of Natural Sciences, Raleigh, North Carolina 27603, USA; 3 Department of Microbiology and Immunology, Montana State University, Bozeman, Montana 59717, USA; 4 Department of Earth Sciences, Montana State University, Bozeman, Montana 59717, USA

## Abstract

Exaggerated cranial structures such as crests and horns, hereafter referred to collectively as ornaments, are pervasive across animal species. These structures perform vital roles in visual communication and physical interactions within and between species. Yet the origin and influence of ornamentation on speciation and ecology across macroevolutionary time scales remains poorly understood for virtually all animals. Here, we explore correlative evolution of osseous cranial ornaments with large body size in theropod dinosaurs using a phylogenetic comparative framework. We find that body size evolved directionally toward phyletic giantism an order of magnitude faster in theropod species possessing ornaments compared with unadorned lineages. In addition, we find a body mass threshold below which bony cranial ornaments do not originate. Maniraptoriform dinosaurs generally lack osseous cranial ornaments despite repeatedly crossing this body size threshold. Our study provides novel, quantitative support for a shift in selective pressures on socio-sexual display mechanisms in theropods coincident with the evolution of pennaceous feathers.

## Abstract (web summary)

Many of the theropod dinosaurs, the group including *Tyrannosaurus rex*, had bony ornamentation on their skulls. Here, Gates *et al*. show that such ornaments are associated with greater body size and accelerated body size evolution in theropods; however, these relationships are absent in the maniraptoriform dinosaurs, which had evolved pennaceous feathers.

## Graphical abstract

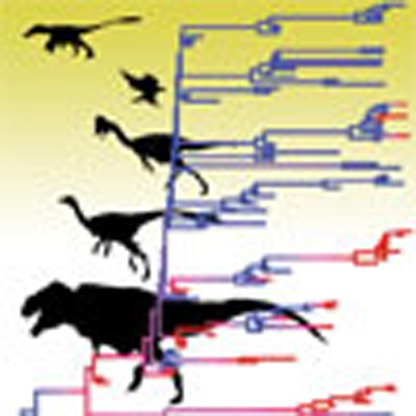

Sexually selected morphological traits represent some of the most ostentatious adaptations known and perform a wide variety of functions, including intimidation, defence and communication^1^,^2^,^3^. The panoply of functions among sexually selected traits has elicited their further differentiation into weapons and ornaments. McCullough *et al*.^4^ define a weapon as a morphological trait present on males that is used in male–male combat, whereas the same authors define an ornament as a trait possessed by males that is the basis of female choice. Use of these definitions in modern organisms is possible because sexually selected traits are observed in action. Fossil species with no living descendants pose a unique problem to defining traits as sexually selected^5^, let alone attempting to further differentiate into weapons or ornaments. Prior studies of fossil traits have used terms such as ‘exaggerated structures'^6^,^7^ or ‘extravagant structures'^5^ to accommodate the uncertain nature of fossil features. In this paper, we refer to fossil exaggerated structures as ornaments, bearing in mind other functional definitions mentioned above.

Most prior research on ornaments has attempted to explain their evolution mechanistically or socially through the lens of individual species. However, in addition to these individual-level effects, ornaments have important macroevolutionary consequences that impacted amniote evolution generally, such as increased speciation rates^8^,^9^,^10^,^11^,^12^, and they can provide critical clues about socioecological and habitat parameters that are otherwise difficult to estimate for extinct species^13^. For example, studies on extant animals infer a strong habitat-mediated evolution and exaggeration of ornaments (including conspicuous coloration) in ungulates^13^,^14^, owls^15^, galliform birds^16^, chameleons^17^,^18^, iguanas^19^ and guppies^20^, suggesting that understanding the evolution of ornaments may offer a valuable mechanism for inferring habitat in extinct species. Exploration of the evolution of socio-sexual ornaments in extant animal groups such as in ungulate mammals^13^ and galliform birds^16^ also demonstrates a correlation with key morphological parameters such as body mass.

The most diverse and extreme array of cranial osteological ornaments of any amniote group, living or extinct, evolved in dinosaurs; yet to date, little is understood about the drivers, socioecological and morphological correlates, and macroevolutionary consequences of these adaptations on speciation, extinction and physiology in this clade. We hypothesize that the association between body mass and cranial ornamentation documented in some extant tetrapods^13^,^16^ should also hold for non-avian theropod dinosaurs owing to their body mass extremes (0.1−>7,000 kg, five orders of magnitude range in intra-clade body mass) and exaggerated cranial ornaments. General observation indicates that 20 of the 22 largest theropod dinosaurs for which skulls are known (for example, *Tyrannosaurus rex*) possess some form of osteological cranial ornament. Conversely, smaller species appear to largely lack such structures, supporting our hypothesis.

Here, we test for a correlation between the presence of extraneous bony ornaments and large body size in non-avian theropod dinosaurs finding evolutionary links between phyletic giantism^21^ (an evolutionary trend toward large size that is already documented for dinosaurs as a whole^22^) and cranial ornamentation. Our results show a sustained directional trend toward giantism only in more basal theropods that possess ornaments, whereas those species that are projected to have possessed pennaceous feathers seem to have achieved a wide range of body sizes without the presence of bony ornaments. This dichotomy in the association between large body and cranial embellishments suggests that the evolution of pennaceous feathers may have shifted visual communication structures away from bony adornments toward feather displays.

## Results

### Bony cranial ornaments are correlated with body mass

**Figure 1:**
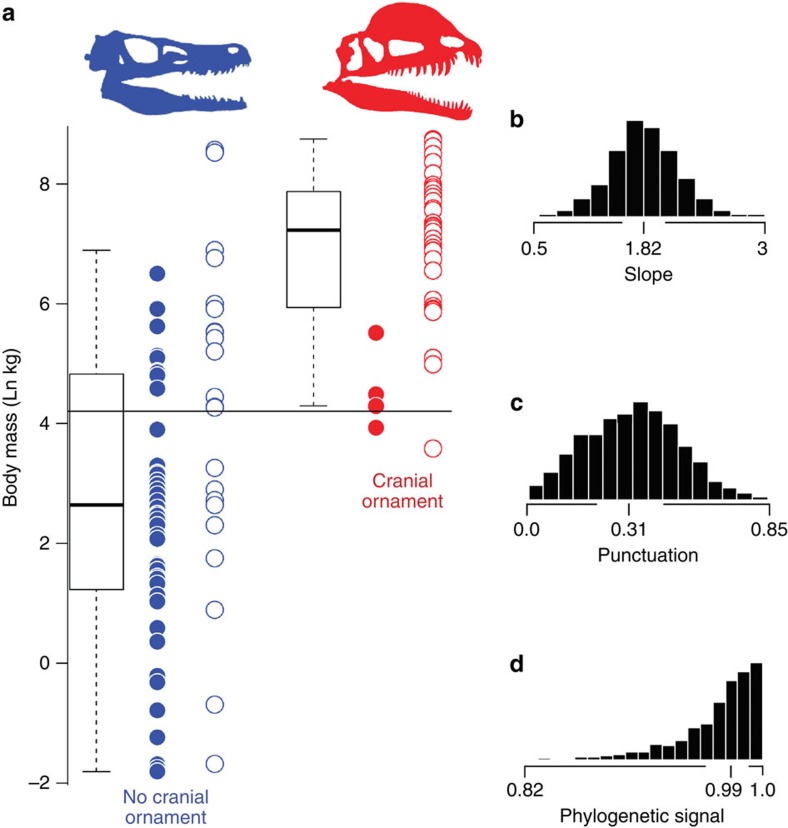
Phylogenetic *t*-test data distribution performed in BayesTraits. (**a**) Blue data points represent unornamented theropods, red represents ornamented species, open circles are non-maniraptoriform theropods, whereas closed circles are species found within Maniraptoriformes, total sample size in phylogenetic *t*-test is 111 theropod species. Horizontal black line shows the mean log_*e*_ body mass (4.207) among all theropods in the sample. Boxes within the box plot shows the first and third quartiles of the data; whiskers expand through the 95th quartile. The estimated posterior distribution for (**b**) slope, (**c**) punctuation and (**d**) phylogenetic signal shown on right.

We tested whether species with cranial ornaments evolved larger sizes relative to species that lack such display features. Using maximum likelihood on a single consensus tree, the best fitting phylogenetic generalized least squares (PGLS) regression model includes the *λ* (phylogenetic signal) and κ (punctuation) parameters, with an Akaike Information Criterion (AIC) weight of 1. We followed this analysis with a Bayesian regression model (phylogenetically normalized *t*-test) where *λ* and κ were both estimated during the Markov-chain Monte Carlo (MCMC), and which sampled over 3,000 time-scaled trees to account for our uncertainty in time calibration. The posterior distribution of the slope parameter, a, ranged from 0.5 to 3 (median=1.83), indicating strong support for the hypothesis that large body size evolves coincidently with cranial ornamentation (phylogenetic *t*-test mean *R*^2^=0.32; Fig. 1). Testing the same model against the null hypothesis for regressions in which the slope is forced to 0 (that is, no correlation), a Bayes factor value of 21.5 indicates very strong support that large theropod dinosaurs evolved cranial ornamentation more often than smaller species. Phylogenetic signal was close to one in all analyses (Fig. 1). We also find low estimates for the κ (punctuation) parameter, with a posterior median of 0.31, suggesting that shorter branches contribute more to trait evolution than longer ones.

Augmenting the results from the PGLS, we took advantage of the discrete character analytical ability of the threshold model of evolution^23^,^24^. The threshold model of evolution is a method adapted by Felsenstein^23^ to calculate correlations between discrete and continuous variables. To accomplish this goal, we assume that a series of unknown, unobserved continuous variables underlies each observed discrete character. In our case, these quantitative biological variables such as metabolic rate, hormone levels, eyesight and so on, all possibly play a role in the appearance of crests through theropod lineages, but are unknown and unattainable. These invisible continuous traits are converted to liabilities by means of a multivariate normal Brownian motion model that randomly, continuously evolves a discrete trait until it passes over a threshold, thereby converting the original discrete character state to the next. Brownian evolution of the trait in question continues throughout the phylogenetic tree until the tips are reached, wherein the estimated liability for each tip is used in a correlation test with the known continuous variable^23^,^24^. Our analysis finds a high correlation between body mass and the presence of cranial ornaments in theropod dinosaurs (threshold model linear regression mean *R*^2^=0.759; Supplementary Table 1).

After establishing a correlation between large body mass and the presence of bony cranial ornaments, we set to estimate the body mass below which bony ornaments are not predicted to evolve. The PGLS analysis from above estimates a minimum of 55.2 kg for this body mass threshold, whereas the lowest estimate is predicted to be 36 kg simply by using the body mass of *Syntarsus kyentakatae*, the smallest ornamented theropod in our sample. A different method of determining the body mass minimum is to use the *θ* (theta) parameter estimated for unornamented theropods in generalized Hansen models^25^ (that is, modified Ornstein–Uhlenbeck model). We found that the best fitting model estimated a large difference between unornamented body mass optima (10.96 kg) and ornamented optima (1,396 kg). One reason for the discrepancy in body mass estimates between the PGLS and the generalized Hansen models is that the latter included data for only the non-maniraptoran portion of the phylogenetic tree (see ‘Methods' section for explanation) and that estimating parameter values for single and mulitpeak OU models is prone to varying amounts of error based on provided data^26^ (see ‘Discussion' section for further information on OU biases).

### Larger body size evolution for adorned theropods

Many lineages, but certainly not all theropod lineages, underwent the morphological transition of gaining or losing osteological cranial ornamentation. We estimated the transition rates at which cranial ornamentation is gained and lost using reversible jump MCMC (RJMCMC), which is a Bayesian method that efficiently explores the possible model parameters without fully exploring the entire model space. One hundred per cent of the posterior distribution is a one-parameter model (where the rate of gaining cranial ornaments is equal to the rate of loss). To test the confidence in the equal rates model derived from the RJMCMC analysis, we also compared a non-reversible jump analysis using an exponential hyperprior (seeds the mean of the exponential prior from a uniform on the interval 0–10 and allowing rates to vary across the parameter estimates) to an equivalent analysis with the rates forced equal to one another. A Bayes factor of 2.5 indicates some support for the equal rates model. Results from the fitDiscrete function in the R package geiger^27^ concurred that an equal rates model best fit the data (although the symmetrical model fit equally well according to this analysis; Supplementary Table 2).

Next, we estimated the rate at which a theropod lineage crosses the 1,000 kg value of giantism among theropods, as defined by Erickson *et al*.^28^, with and without cranial ornamentation. An RJMCMC analysis suggests that discretized body mass (≥1,000 kg) evolved an order of magnitude faster ( × 20 faster) in lineages possessing cranial ornaments (average posterior transition rate to large body size in species lacking ornaments=0.01, in species with ornaments=0.2). These data indicate that body mass evolution across the 1,000 kg threshold in non-avian theropods was dependent on the prior acquisition of ornaments.

Generalized Hansen models provided similar results as RJMCMC. However, using the entire theropod tree produced parameter outcomes that were beyond biological reality, most likely because of conflicting patterns of crest development and large body size evolution among the maniraptorans^29^ compared with more basal species. For instance, *α* parameter values, those designating the attraction of lineages toward an optimum, were consistently estimated at exceedingly low values of 10^−9^ and *σ*^2^ values of evolutionary rate were estimated at low rates of 10^−2^. In addition, estimates of the two body mass optima, *θ*, for ornamented species were in many cases approaching 50% higher than the largest theropods in the database. Estimating the root *θ* produced even worse results with values well beyond reason (for example, exp[10^−6^] body mass for unornamented species and exp[10^7^] body mass for ornamented species, with standard errors larger than the estimated *θ* values). Since we were most interested in the effect of bony cranial ornaments on the evolution of body size, we trimmed the theropod tree to exclude maniraptoriform species thereby removing noise from the dataset. As such only the results incorporating the non-maniraptoran portion of the theropod tree are presented here (57 total taxa, 21 unornamented and 36 ornamented).

The best fitting generalized Hansen model optimizations calculated over non-maniraptoran species overwhelmingly supported an OUMV model (Supplementary Tables 3,4; Supplementary Data 1,2), which is one where the body mass parameter estimate *θ* and rate parameter, *σ*^2^, varied across lineages, whereas the *α* parameter stayed constant (Supplementary Tables 3,4). Two optimal peaks of log_*e*_ body mass between ornamented (7.241±0.334, root not estimated^30^) and unornamented (2.394±1.538, root not estimated^30^) theropods show that there is considerable difference between the ornamentation regimes. A single alpha rate parameter (*α*=0.131±0.049) describes how rapidly a lineage is hypothesized to evolve from the lower body mass optimum to the next or if a lineage begins away from the optimum this parameter estimates the pace that the lineage will move toward its optimal body mass. This best fit model also includes a rate parameter, *σ*^2^, that estimates the degree to which body masses vary over lineage evolution between the two optima. These results held with both the root estimated and non-estimated models. The standard error for *θ* in the unornamented regime is considerably higher than for the ornamented regime. This finding is not surprising given that both small and large species are unornamented. Since the vast majority of species that lack bony cranial ornaments are small bodied, the overall *θ* model averaged estimate for log_*e*_ body mass (2.394) is much lower than the log_*e*_ (7.241) model averaged estimated for ornamented lineages. The *σ*^2^ is much larger for unornamented lineages, meaning that jumps between body masses are larger and more sporadic than that in ornamented lineages. The latter regime appears to display a directed trend of body mass evolution, rapidly approaching the log_*e*_ (7.241) body mass once a lineage obtains ornamentation. The highly variable *σ*^2^ parameter (evolutionary rate; *σ*^2^=1.358±0.642) among unornamented lineages compared with the quite small *σ*^2^ (0.313±0.117) for ornamented lineages indicates a directional trend toward phyletic giantism among ornamented theropod clades. An *α* parameter that is relatively small as in our data, combined with a high optima, produces the directional trend pattern within an Ornstein–Uhlenbeck context because once the *α* parameter approaches zero, OU models reduce to Brownian motion with a trend^31^. Although the *α* parameter is not extremely close to zero, when combined with the low *σ*^2^, together they can produce the same evolutionary result of directional evolution toward the optima^25^.

Albeit only a rough approximation^25^,^31^, using the alpha parameter to calculate the phylogenetic half-life^31^,^32^ predicts that once bony ornaments are acquired in a theropod lineage, body mass could have evolved halfway toward the gigantic optimum about every 5.291 Ma. Whereas this rate may not reflect true biological reality^25^, the predicted phylogenetic half-life is only 3% of the entire tree age, indicating rapid evolution among theropods into two optima instead of a single optimum with a trend. That is, this model suggests that once a lineage gained ornaments, body mass evolution became more directed with smaller lineages quickly attaining gigantic proportions toward the ornamented optima.

### Cranial ornamentation evolved independently

We also evaluated the hypothesis that osteological cranial ornamentation evolved convergently in different theropod lineages by comparing analyses in which nodes are ‘fossilized' to a given state. A Bayes factor test of 9.4 provides strong evidence that the ancestral theropod lacked cranial ornamentation, and therefore this trait evolved convergently in different groups of theropods. Our analyses further suggest that cranial ornamentation is ancestral for Allosauria^33^ (*Allosaurus* and kin; Bayes factor test=6.5), but the ancestral condition in Tyrannosauroidea (*Tyrannosaurus* and kin) is only weakly supported (Bayes factor test=2.5 favoring ornamentation), despite the two most primitive members of the clade possessing ornamentation. The analysis suggests that the ancestor of *Dilong* (which lacks ornaments) and *Tyrannosaurus* lacked cranial ornamentation (Bayes factor test=11.3). Among more derived tyrannosaurs, it is not until the ancestor of *Appalachiosaurus* and *Tyrannosaurus* that we find strong evidence for cranial ornamentation (Bayes factor test=15.3).

In an effort to posit hypotheses of causality, we assessed whether cranial ornamentation or large body size evolved first in different groups of theropods. We suggest that the common ancestor of Allosauria^33^ was large bodied (Bayes factor test=8.2 for binary analysis of body mass using RJMCMC, with a log_*e*_ body mass of 7.3 using Brownian motion continuous models) and as noted above possessed cranial ornamentation. Our analysis suggests that the tyrannosauroid ancestor of *Dilong* and *Tyrannosaurus* was not large bodied (Bayes factor test=7.7 for binary analysis of body mass using RJMCMC, with a log_*e*_ mass of 4.58 using Brownian motion continuous models). Paralleling results above, large body size, like cranial ornamentation, does not evolve in tyrannosauroids until the common ancestor of *Appalachiosaurus* and *Tyrannosaurus*, (Bayes factor test=7.3 for binary analysis of body mass using reversible jump MCMC, with a log_*e*_ mass of 6.91 using Brownian motion continuous models); however, the basal tyrannosauroids *Proceratosaurus* and *Guanlong* do possess cranial ornaments.

Given the controversy of ontogenetic maturity in the only known specimen of *Raptorex*^34^ and the known skeletal immaturity of *Dilong*, our tyrannosauroid results may be slightly skewed because of coding these larger species with no cranial ornaments. Despite the fact that the adult body size is unknown for *Dilong* and *Raptorex*, if we suppose they follow the pattern of most every other tyrannosauroid by possessing cranial ornaments at maturity, their larger mature body size would only increase the fit of our model that bony cranial ornamentation is linked to large body size evolution in non-maniraptoran theropods. Contrary, if their body size remained close to the currently observed size, yet they grew cranial ornaments rapidly at a later ontogenetic stage than preserved, this would lower the slope of the regression line between the unornamented and ornamented body masses, moreover, it would lower the estimated threshold for the attainment of cranial ornaments (*Dilong* is smaller than the presently smallest ornamented species *Syntarsus kyantakatae*). However, the overall effect would likely be minimal provided that these are two taxa out of the whole sample. Nonetheless, ontogenetic uncertainty should be an area of consideration when interpreting the evolution of body size within Tyrannosaurioidea. An additional observation that may have a bearing on the supposed lack of ornamentation in *Xiongguanlong* and possibly *Raptorex* and *Dilong* is that ornamentation style shifted from thin elongated parasagittal crests in basal species *Guanlong* and *Proceratosaurus* to rugose knobs in *Appalachiosaurus* and kin.

### Taxonomy does not affect model results

Maniraptoriform theropods have a noticeable lack of cranial ornamentation compared with more basal species, and comparing their inclusion in Generalized Hansen models to those withholding them from the same models suggests that a different evolutionary trajectory may be at play. PGLS was used to better define a predictive body mass threshold for species required before the acquisition of osteological cranial ornamentation, except in this instance we controlled for the effect of being a maniraptoriform on model predictions of body mass by including an interaction term. Using only non-maniraptoriform theropods produced a regression model of BM=1.98+2.09(Orna), (PGLS mean *R*^2^=0.32, *ɛ*=0.253) where Orna is the presence (1) or absence (0) of osetological cranial ornamentation. Next we tested nested models of the entire 111 species dataset. The simpler model produced a regression equation of BM=1.97+2.04(Orna), (PGLS mean *R*^2^=0.199, *ɛ*=0.395). The more complex model that included the dummy variable and interaction term produced a model of BM=1.97+2.15(Orna)−1.25(Phylo)−0.69(Phylo)(Orna), (PGLS mean *R*^2^=0.216, *ɛ*=0.394), where Phylo refers to either being included within (1) or excluded (0) from Maniraptoriformes. A Bayes factor test of 0.307 shows insignificant difference between the simpler versus more complex model. This means that simply being a maniraptoriform does not increase the model's correlative power, and therefore synapomorphies of the entire clade (for example, presence of pennaceous feathers) do not seem to hold much role in body mass evolution for this clade. It should be noted that prediction via PGLS can be difficult and these body mass predictions should be used as a generalization of the true evolutionary model.

Comparison of the models incorporating the entire phylogeny versus only the more basal taxa shows a much better correlation for non-maniraptoriforms. In large part this is because maniraptoriform species vary more widely in body mass and almost exclusively do not possess osteological cranial ornamentation. Also, the two terms within the more complex model show that body mass within the maniraptoriform clade is generally much smaller than predicted for the basal taxa, and when ornamented maniraptoriform body masses are predicted, the estimate is considerably lower than any predicted for more basal ornamented taxa.

Finally, we performed an ANOVA that controls for the non-independence of theropod dinosaurs in BayesTraits for log_*e*_ body mass, crests (presence/absence) and clade (Maniraptoriformes/non-Maniraptoriformes) to see if there was a difference in the body masses between crested and non-crested forms outside of simply being ornamented. We find strong evidence for differences in body size related to crests (100% of the posterior slope was above 0), yet no evidence for differences in body size related to clade (49% of the posterior was above 0). We also find no evidence for differences in body size related to the interaction between crests and clade (41% of the posterior was above 0).

## Discussion

Our analysis finds a significantly positive correlation between large body mass (log_*e*_) and the evolution of osteological cranial ornamentation in theropod dinosaurs. Moreover, we detect a minimum body size threshold for the acquisition and retention of osteological cranial ornaments that is required to achieve the positive slope correlation between body mass and cranial ornamentation in our sample. Our estimation of the body mass threshold should be taken with some care, as the sample size of ornamented species is relatively low (*n*=38). Increasing the sample of ornamented theropods will better refine the threshold as future discoveries are incorporated. Such a physiological barrier for the evolution of bony ornamentation has not yet been documented in other amniote clades, nor has a link between mass and ornamentation yet been recovered across geologic time scales.

Not only is there a positive correlation between ornamentation and large body mass among theropods, we also recovered the first evidence for accelerated body mass evolution in dinosaurs relating to socio-sexual signalling structures. An increasing number of studies find atypically sustained, accelerated rates of body size evolution among maniraptorans, suggesting unique evolutionary drivers^35^,^36^ such as locomotion (that is, flight^37^); although the evolution of diet seems to have played an ambiguous role^29^,^38^. The recovery of a 20-fold rate increase in crossing the 1,000 kg threshold for phyletic giantism demonstrates that the evolution of theropod body size encompasses socio-sexual dynamics in addition to the ecological/bauplan factors implicated for accelerated rates of theropod evolution^36^,^37^. We further show that not only do theropods with ornamentation cross the giantism threshold more often than unornamented theropods, but results from our generalized Hansen models support the notion that once a theropod lineage obtains osteological cranial ornamentation, the lineage will progress on a directional trend toward phyletic giantism at a more sustained pace than unornamented lineages. Such an insight has not been documented for other vertebrate clades, and therefore may be another important component to consider within socio-sexual signalling. Still to be tested are the relationships between accelerated body mass evolution, speciation rate and extinction rate. The latter two phenomena are proposed to be intimately linked across macroevolutionary timescales^39^, yet the role of increasing body mass via a socio-sexual signalling regime within speciation and extinction rates is complicated and deserves analytical attention.

Species morphology, such as body mass, is a result of environmental and socio-sexual selection pressures jointly guiding organismal phenotypes through time. As such, the relationship between habitat preferences and the expression of socio-sexual traits must be considered alongside patterns of body mass evolution in varying habitats. Manifestation of socio-sexual traits among different species is complicated by many environmental factors that may be useful in predicting the habitat preferences of extinct animals. Among ungulates, one study found that both males and females become larger through evolutionary time and visually more obvious to others in their habitat as a means to maintain a level of conspicuousness. Stankowich and Caro^13^ derived a conspicuousness metric to reflect the relationship between habitat openness and height of animals, whereby a large animal living in dense forest is not conspicuous, a small animal living in open habitat is moderately conspicuous, and a large animal living in open habitat is quite conspicuous. Given the similar selective pressure of maintaining conspicuousness attributed to social system and habitat openness on the visibility of cranial ornaments in modern analogues, we hypothesize (although currently untestable) that (1) many bony ornamented theropods lived in open habitats and (2) the socio-sexual system that rewarded bony ornaments and an open living habit were likely dual drivers of the mass increases in theropod dinosaurs included in our analyses. These evolutionary hypotheses are generalizations and do not include lots of ecological drivers such as predation pressure, available land area, and ecological release. For example, Hone *et al*.^40^ hypothesized that large theropod lineages would be under selection pressure to reduce visibility during predation, Sampson and Loewen^41^ and Loewen *et al*.^42^ hypothesized that tyrannsaurids evolved body size in relation to relative seal level change, Zanno and Makovicky^43^ hypothesized that North American tyrannosaurids only began evolving large body size after the extinction of giant allosauroids.

Although such a hypothesis for the macroevolutionary trend seen in bony ornamented dinosaurs is consistent with data from extant megavertebrates, other selective pressures seem to drive trends differently in some small-bodied taxa. Many small, ornamented amniotes exhibit visual cues consistent with an effort to decrease predation pressure while simultaneously maximizing visibility to conspecifics. For instance, chameleon populations that reside in open habitats are smaller bodied than their closed habitat populations, and their cranial ornaments are relatively smaller and duller in colour^18^. Birds, chameleons and iguanas living in closed habitats have brighter plumage^44^, scales and casques^18^, and dulaps^19^,^45^, respectively. Thus, in these examples, there exists an overarching trend to retain the visibility (and thus display function) of ornaments in open habitats, even when there is selective pressure toward smaller body sizes presumably to counteract predation pressure^18^. Some smaller theropods, such as *Caudipteryx*, utilized tail displays that may have provided an alternative to cranial display with the purpose of maximizing visibility with small body size.

Differentiating the effect of organismal body size on macroevolutionary trends of ornamentation has not been widely examined across taxonomic groups. All of the vertebrates mentioned above that deviate from the dinosaurian and ungulate trend are relatively small bodied, needing in part to balance socio-sexual selection pressure with that of predation selection pressure^18^. Our preliminary evidence suggests that body size may have a threshold with which smaller ornamented organisms maintain an optimal adaptive peak for smaller size in open habitats whereas larger ornamented vertebrates are afforded a more rapid evolution toward a unique adaptive peak of large body size. This hypothesis stems from observed ecological trends of small-bodied species being small in part to presumably avoid predation risk in open habitats whereas larger vertebrates have been shown to increase in size in the same open environments through several possible selective factors. More in-depth work is needed with models of stabilizing selection^25^ and hidden Markov models^46^,^47^ to gain a fuller understanding of the adaptive landscape that underpins body size evolution in open versus closed habitats.

One final unknown behaviour that could affect the presence of ornamentation and possibly body size in theropods irrespective of habitat is the utilization of leks as a means for reproductive showcases. Recent evidence suggests some theropod species participated in lekking^48^, meaning that open arenas in closed habitats could have evolutionary outcomes similar to an open living habitat.

**Figure 2:**
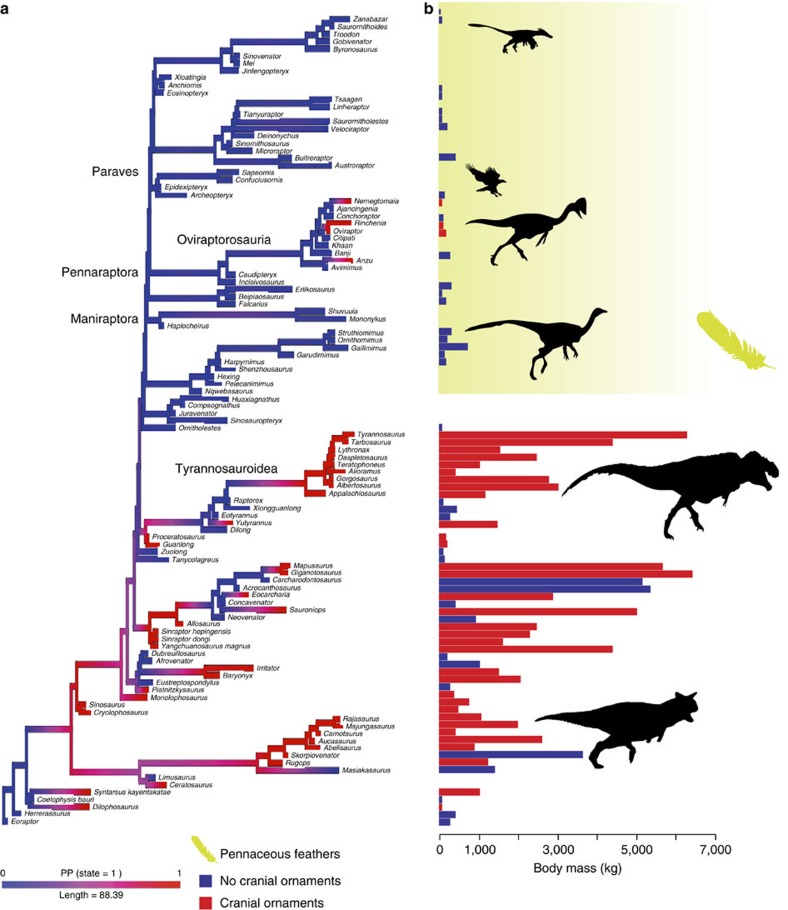
Phylogeny of non-avian theropods used in this study. (**a**) Density map of Bayesian stochastic character probabilities of unornamented (blue) and ornamented (red) character states. (**b**) Body mass estimates for non-avian theropods used in this study. Green shading shows the distribution of pennaceous feathers among theropods.

The widespread presence of bony cranial ornaments in basal theropods stands in marked contrast to the near absence of this trait in Maniraptoriformes (theropods most closely related to, and including, birds; Fig. 2). We find that 13 maniraptoriform species exceed the PGLS predicted body mass for ornamented theropods (55.2 kg), yet few evolved osteological cranial ornamentation (Figs 1 and 2). This observation is unlikely to derive from an evolutionary trend toward lightened skeletons for powered flight because only one clade of maniraptorans achieved this locomotion and several others achieved very large proportions. All taxa that did develop osteological cranial ornamentation belong to a single clade—Oviraptorosauria—raising intriguing questions about the habitat and socioecology of this clade. We tested the significance of this observation by performing an ANOVA for body mass and bony crest presence while controlling for the phylogeny. We find that maniraptoriforms are not significantly different in body size compared with more basal theropods and that there is no difference related to body size in large bodied species in both groups that lack crests. The low frequency of bony ornamentation in large bodied maniraptoriforms contrasts with an ornamentation rate of 75% in non-maniraptoriform taxa that exceeded the mass threshold, provoking an explanation. We find these data most consistent with the hypothesis that the evolution of pennaceous feathers pivoted signalling strategies for maniraptoriform theropods away from osteological structures^40^, toward soft tissue structures including feathered and soft tissue crests as now documented in several maniraptoriforms^49^,^50^.

Both osteological and feather displays serve as signalling structures; however, they vary both physiologically and functionally^40^. Feathers originate from a single follicle in the dermis of theropods, producing a three dimensional structure that can act alone or in concert with other plumage to produce a wide range of displays^51^. Whereas, osteological features form by accelerated growth of bone on various regions of the skull overlain by an assortment of tissues^52^,^53^. Another facet of feather usage in display structures is the ability in modern birds to change the size and colours of their feathers through muscular control of surrounding tissue and by seasonal moulting or abrasion^51^, respectively. In contrast, once formed, osteological ornamentation is consistently on display (although colour changes can occur in beaks of some birds throughout the year, for example, puffins^54^). The implication of permanency in osteological structures has ripple effects that carry from early development throughout the life of the ornamented individual. Shifting from bony ornamentation perennially on display to that of ephemeral feathers could have diminished the problem of early life deficiency. Modern studies have shown that poor resources in early life development can be reflected in diminished quality of bony social signalling structures throughout the remainder of life^55^, whereas the moulting of feathers allows for an honest reflection of quality at the time of signalling. Canonical studies of theropod life history demonstrate that the largest theropod species achieved giantism by means of accelerated growth rates early in life^28^ and that sexual maturity (and the likely investment in sexual signals) preceded asymptotic growth^56^. Any reduction in resources early in the development of ornamented theropods may have reflections in ornament quality throughout the life of the animal, whereas dinosaurs utilizing pennaceous feathers for display would have been able to compensate via new reproductive displays each moult, although it should be noted that the effect of this mechanism decreases markedly as size of the theropod increases and the ornamental structure becomes relatively smaller.

Factors prompting the early evolution of pennaceous feathers in extinct taxa are difficult to quantify. A socioecological dimension to the selective forces acting on pennaceous feathers, as documented here and as proposed by studies of coloration^49^, provides additional context for the appearance of pennaceous feathers before the evolution of flight^57^, and indicates that socio-sexual behaviours observed in extant birds may have shaped the early evolution of integumentary displays in their extinct theropod ancestors.

## Methods

### Phylogenetic tree building

A phylogenetic tree of 111 theropod species that preserve adequate skull elements for the detection of cranial ornamentation (Supplementary Fig. 1; Supplementary Table 5) was grafted based on the topologies from a variety of sources for specific clades. To account for the uncertainty in stratigraphic and temporal placement of theropod specimens^58^, we scaled 1,000 trees each using three methods available in the R package paleotree v.2.0 (ref. 59). Time-scaled trees were produced using the methods ‘All Branches Additive' (ABA), which adds one million years to each branch, ‘Minimum Branch Length' (MBL), which in our case scales all branches to at least one million years then takes away time from earlier branches to give to later branches, and ‘Equal', which attempts to equilibrate the time distributed on the tree by adding time to the root of the tree and then adjusting zero-length branches by borrowing time from earlier branches to give to later branches^59^. Time intervals created for the time interval matrix are listed in Supplementary Table 6. The most accurate geologic times available for each theropod taxon were used in the creation of intervals. Vartime equalled 1 in all scalings. For Bayesian analyses, we accounted for branch length uncertainty^58^ by sampling 1,000 trees for each time calibration method noted above (Equal, ABA and MBL) during the MCMC process. A consensus tree was created from all 3,000 trees to run in maximum likelihood analyses.

### Cranial ornamentation coding

Cranial ornamentation, a two-state discrete character, was attributed to theropod species that exhibited bone features that differed from typical shape and texture of skull elements including sagittal and parasagittal crests, horns, knobs and rugosities. The rugosity observed on abelisaurid skulls was not scored as an ornament in an effort to detect ornamentation signals of those abelisaurid taxa with other styles of ornamentation. In addition, Carrano and Sampson^60^ identified the facial sculpturing abelisaurids as likely a homolgous, unambiguous synapomorphy of Abelisauridae. The inclusion of this trait within our coding would not provide the breadth of character distribution we sought for our comparisons, in addition to being ubiquitous across all sampled abelisaurids. Many abelisaurid species, however, do possess further bony structures such as horns (for example, *Carnotaurus*) and bosses (for example, *Majungasaurus*). As such all possible abelisaurid species were coded for the presence or absence of these traits. Other clades, such as Tyrannosauridae, have a single ornamental trait nearly ubiquitous across all taxa (in this case that of rugosities and bosses), yet this same trait is seen across other theropod clades and was retained in the analysis. Of the 111 taxa sampled for this study 38 were coded as possessing ornamentation. A potential source of bias originates from the fact that one needs only a particular ornamented skull piece to code a taxon as ornamented, whereas a nearly complete skull would be required to definitely code as absent. To decrease bias toward ornamented skulls, we coded taxa as unornamented (for example, *Falcarius utahensis*) if a specimen preserved ‘plain' skull elements that would otherwise generally display ornamented structures (nasals, postorbitals, frontals) in closely related taxa, even if not all of the skull was present. However, there is still a possible bias toward ornamented theropods in our dataset. Future discoveries may add to the number of ornamented species in this study currently coded as unembellished. Ontogenetic stage represents another potential bias in the dataset. The only specimens of *Juravenator* and *Dilong* are skeletally immature, and some contend that *Raptorex* falls within the same ontogenetic category^34^. However, theropod dinosaurs achieved reproductive maturity before skeletal maturity, therefore socio-sexual signalling traits such as cranial ornamentation are likely to have evolved before skeletal maturity^56^, reducing this potential bias in the sample. We therefore coded these species as they are preserved since we do not know the morphology at maturity. Further discussion of these ontogenetic considerations is given below where applicable, but in short, if these small theropod species are unornamented at maturity it supports our results and if ornamented but small body sized at maturity they will be outliers to the current data although likely will minimally affect our results in the opposite direction because these are three species out of 111 (or 58 in the OU models).

### Body mass estimation

Body masses were estimated using the femoral length equation of Christiansen and Farina^61^ with data sourced mostly from Zanno and Makovicky^29^. Specimens that did not possess a femur and not included in Zanno and Makovicky^29^ were estimated by scaling a skeletal element and body mass from a closely related taxon or taken from other sources (Supplementary Table 5). All body masses were log_*e*_ transformed before analyses.

### Cranial ornamentation and body mass correlation tests

We used the programme BayesTraits (http://www.evolution.rdg.ac.uk) to analyze body size and cranial ornament data (Fig. 1) in both maximum likelihood and Bayesian frameworks.

To begin we tested correlation between the continuous character of log_*e*_ body mass and the discrete cranial ornamentation character using the threshold model within the threshBayes function of phytools v. 0.3-72 (ref. 62). The threshBayes analysis was run for 1,020,000 generations, using default prior, liability, and other parameters. For further evolutionary analysis we created posterior distributions of phylogenetic generalized least square (PGLS) regression models. This method accounts for the evolutionary non-independence among the characters^63^,^64^ by transforming the residuals with a phylogenetically derived variance-covariance matrix. Cranial ornamentation was treated as the independent character, which allows us to test whether a significant difference in body size evolved between species that have or lack cranial ornamentation (phylogenetic *t*-test). Hypothesis testing was performed by comparing results from the above method with one in which the slope was forced to 0 using a Bayes Factor test.

The scaling parameters *λ* (phylogenetic signal), κ (punctuation), *δ* (time of change) were sampled during the MCMC regression analysis two at a time (*λ*, κ and *λ*, *δ*), which produced posterior distributions of regression models. Phylogenetic signal (*λ*) scales the off-diagonal elements of the variance-covariance matrix^63^,^65^. *λ* ranges from 0 (the data do not vary according to the tree) to 1, where character variation is predicted by the phylogeny. Acceleration (*δ*), scales the matrix with an exponential transform while punctuation (κ) is an exponential transform applied to all branches in the tree. We ran the analysis for 1,010,000 iterations sampling every 1,000 iterations with a burn-in of 10,000. The default (and non-assuming) uniform prior (from −100 to 100) was used.

Testing for a relationship between body mass and the presence of cranial ornamentation with and without maniraptoriformes involved the use of a PGLS regression excluding all maniraptoriform species as well as a second analysis that included an interaction term. Within the second analysis in addition to the dependent variable—log_*e*_ body mass—and the independent variable cranial ornamentation presence (1) or absence (0), we included a dummy variable designating if taxa were maniraptorans (0=non-maniraptorans, 1=maniraptoran), and an interaction term, which is the product of the independent variable and the dummy variable. We ran the analysis for 5,050,000 iterations sampling every 1,000 iterations with a burn-in of 50,000. The default (and non-assuming) uniform prior (from −100 to 100) was used. *λ* was estimated throughout the model. Model assessment was evaluated based on Bayes Factor test (see below).

### Ancestral state reconstruction

The ancestral state reconstruction density map^66^ in Fig. 2 was constructed in phytools v.0.4-45 (ref. 62) using a stochastic map of 10,000 generations and the ‘SYM' (Symmetrical) model of evolution on the time-scaled consensus tree. Further testing of ancestral states was accomplished using Bayes factors where certain nodes were ‘fossilized' to different character states in BayesTraits then compared with the trees with the nodes ‘fossilized' to the opposite state.

### Evolutionary transition rates test

To investigate the rate at which ornamentation is gained and lost we used the RJMCMC discrete method^67^,^68^. The reversible-jump algorithm produces posterior distributions for the rates of changes, and automatically finds the models with the fewest number of parameters by setting rates equal to one another or setting them to zero. An exponential hyperprior for the transition rates was used (the mean of the exponential prior was seeded from a uniform on the interval 0–10). We assume that stationary frequencies of the character states are equal to their observed (empirical) frequencies. The Markov chains were run for 2,010,000 iterations and sampled every 1,000 iterations following a 100,000 iteration burn-in. We evaluated the results from this analysis by comparing results with two non-reversible jump analyses where one was left to procure rates on its own and the other was forced to have equal rates. A Bayes factor test was used to evaluate the two Bayesian posterior distributions.

In addition, we ran the fitDiscrete function in the R package geiger v.2.0.3 (ref. 27) as a second test of ornamentation evolutionary rate. The binary trait, ornamentation, was tested using equal rates, symmetrical rates, and all rates different models with no transformation of branch lengths. Model fitting was assessed via AICc and Akaike weights.

Further rate testing was accomplished through generalized Hansen models (modified Ornstein–Uhlenbeck models of continuous trait evolution under selective regimes)^31^ using R version 3.1.1 and the package OUwie v.1.43 (ref. 25). OUwie results on the whole tree produced nonsensical results (see ‘Results' section where we discuss generalized Hansen models at length), therefore, we trimmed the tree to include only those taxa below the Maniraptoriformes node (that is, defined on our tree as species exclusive of the most recent common ancestor of *Ornithomimus* and *Zanabazar*). OUwie analyses were run over all available models across 25 SIMMAP-style trees produced from the consensus time-scaled tree in phytools^62^ v. 0.4-56 using an Equal Rate model of mapping (chosen based on the results of both the RJMCMC and fitDiscrete analyses). Regimes were designated simply at presence (1) or absence (0) of cranial ornamentation. Body mass was the continuous trait. Analyses were run with both the root optima (ancestral state; root.station) estimated and not estimated. Code for all R analyses is available from TAG on request.

### Model evaluation

We evaluated hypotheses with the AIC to select among regression models with different branch length scaling parameters. The AIC is defined as: AIC=−2 (log likelihood)+2*K*, where the likelihood is the probability of the data given a model and *K* is the number of free parameters^69^. When calculating AIC values, we used a bias-adjustment for small sample sizes as follows: AICc=−2 × ln(likelihood)+2 × K+(2 × K × (K+1))/(*n*−K−1). In either case, the model with the smallest AIC is the preferred model. ΔAIC (Δ_*i*_) for each model is the difference between the AIC of the best model (smallest AIC) and each model's AIC. To choose among models Akaike weights (w_*i*_) are calculated as w_*i*_=exp(−Δ_*i*_/2)/Σ(exp(−Δ_*i*_/2)). Likelihood ratio tests and AICc tests were conditioned on a single consensus tree. Bayesian hypotheses were evaluated using Bayes factors^70^, defined as BF (log)=2 × (log[harmonic mean(complex model)]−log[harmonic mean(simple model)]). Bayes factors over 5 indicate strong support, and values over 10 indicate very strong support.

### Data availability

The data and R code that support the findings of this study are available in the supplementary information files and from the corresponding author upon request.

## Additional information

**How to cite this article:** Gates, T. A. *et al*. Bony cranial ornamentation linked to rapid evolution of gigantic theropod dinosaurs. *Nat. Commun.* **7,** 12931 doi: 10.1038/ncomms12931 (2016).

## Supplementary Material

Supplementary Information Supplementary Figure 1, Supplementary Tables 1-6, Supplementary Discussion, Supplementary Methods, Supplementary References

Supplementary Data 1 All model results from the OU analysis with the root estimated.

Supplementary Data 2 All model results from the OU analysis with the root not estimated, but instead taken from the status distribution.
