# Supplementary material for: Bony cranial ornamentation linked to rapid evolution of gigantic theropod dinosaurs
Source: Nat Commun. 2016 Sep 27;7:12931. doi: 10.1038/ncomms12931 (PMC5052652; doi:10.1038/ncomms12931)
Supplement: Supplementary Information — Supplementary Figure 1, Supplementary Tables 1-6, Supplementary Discussion, Supplementary Methods, Supplementary References [file ncomms12931-s1.pdf]

Supplementary Figures

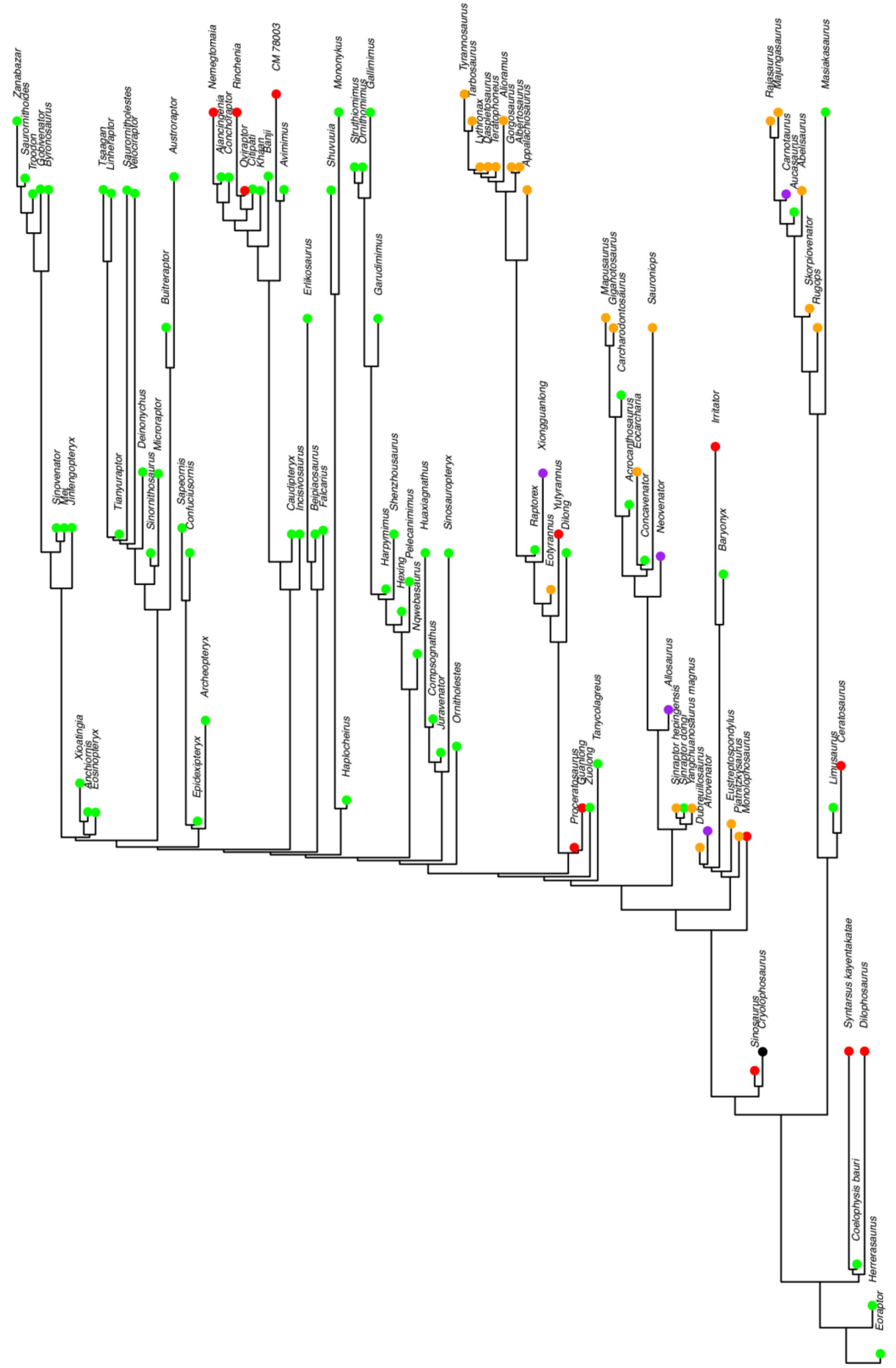

Supplementary Figure 1. Time calibrated phylogeny of 111 theropods used in this study. Colors represent cranial ornamentation: green, absent; red, parasagittal crests; black, forehead crest; purple, brow horns; orange, bumps and rugosities.

Supplementary Tables

Supplementary Table 1. Summary of threshBayes results

	Gen	Sig 1	Sig 2	a1	a2	r	Logl
Min	0	1	0.142	-12.438	0.779	-0.065	-543.3
1st Qu.	255000	1	0.214	-6.795	1.925	0.571	-509.8
Median	510000	1	0.243	-5.367	2.292	0.650	-502.2
Mean	510000	1	0.247	-5.359	2.291	0.631	-502.8
3rd Qu.	765000	1	0.272	-3.875	2.661	0.711	-495.5
Max	1020000	1	0.473	1.966	4.201	0.871	-465.4

Gen, number of generation; 1st Qu, first quarter of the analysis; 3rd Qu, third quarter of the analysis.

Supplementary Table 2. fitDiscrete results of ornamentation evolutionary rates tests

	Equal Rates	Symmetrical	All Rates Different
q12	0.016	0.016	0.016
q21	0.016	0.016	0.026
Lnl	-54.09	-54.09	-53.515
k	1	1	2
AIC	110.18	110.18	110.03
AICc	110.216	110.216	111.141
Weight	0.38	0.38	0.24
RW	1.59	1.59	

q represents the rate of transitioning from one state (1 or 2) to the other. Lnl is the log likelihood of the model. k is the number of parameters in the model estimation; and AIC and AICc represent the Akaike Information Criterion and the sample size corrected AIC score. Rw is the AICc relative weight of the best fitting model compared to the second best model

Supplementary Table 3. Model estimates of AICc selected Generalized Hansen Models from OUwie with root not estimated.

Tree	Model	Log Lik	AICc	Theta Root	Theta0	Theta0 s.e.	Theta1	Theta1 s.e.	α0	α0 s.e	α 1	α 1 s.e.	$\sigma^2$0	$\sigma^2$0 s.e.	$\sigma^2$1	$\sigma^2$1 s.e.	Akaike Weights
1	OUMV	-88.959	191.564	3.292	2.06	1.863	7.058	0.144	0.158	0.035	0.158	0.035	1.776	0.719	0.15	0.046	0.98803
2	OUMV	-98.095	209.837	3.441	2.458	1.174	7.137	0.311	0.088	0.051	0.088	0.051	0.856	0.447	0.162	0.09	0.48532
3	OUMV	-102.124	217.896	3.327	2.279	0.923	7.303	0.912	0.037	0.017	0.037	0.017	0.603	0.26	0.137	0.047	0.50093
4	OUMA	-99.652	212.951	4.589	2.061	0.871	8.029	0.29	0.062	0.02	0.053	0.019	0.495	0.154	0.495	0.154	0.48142
5	OUMV	-98.149	209.944	1.427	2.874	2.695	7.085	0.199	0.213	0.06	0.213	0.06	3.275	1.482	0.233	0.085	0.99557
6	OUMV	-99.365	212.377	2.426	2.685	1.148	7.5	0.474	0.079	0.031	0.079	0.031	0.894	0.378	0.168	0.068	0.65747
7	OUMVA	-90.842	197.924	2.896	2.498	1.373	7.208	0.428	0.072	0.022	0.083	0.021	1.237	0.53	0.019	0.016	0.84298
8	OUMV	-89.781	193.208	0.001	3.598	1.368	7.43	0.179	0.103	0.027	0.103	0.027	1.09	0.422	0.109	0.039	0.99191
9	OUMV	-101.995	217.637	3.555	2.337	1.343	7.12	0.494	0.127	0.093	0.127	0.093	1.032	0.837	0.302	0.163	0.41575
10	OUMV	-94.369	202.385	2.637	2.503	1.288	6.938	0.38	0.114	0.051	0.114	0.051	0.974	0.48	0.199	0.087	0.69203
11	OUMV	-99.252	212.151	2.732	2.369	2.306	7.069	0.243	0.21	0.059	0.21	0.059	2.41	1.023	0.356	0.128	0.96487
12	OUMV	-102.448	218.543	3.807	1.861	2.061	6.92	0.306	0.194	0.07	0.194	0.07	2.016	0.906	0.408	0.167	0.61504
13	OUMV	-96.687	207.022	2.907	2.617	1.201	7.39	0.229	0.1	0.044	0.1	0.044	0.856	0.37	0.178	0.088	0.74887
14	OUMV	-93.113	199.873	2.784	2.52	1.875	7.695	0.17	0.166	0.033	0.166	0.033	1.782	0.659	0.173	0.057	0.78463
15	OUMV	-97.145	207.938	2.582	2.436	1.947	6.993	0.294	0.174	0.081	0.174	0.081	1.959	1.12	0.256	0.129	0.99668
16	OUMV	-96.281	206.209	1.355	3.085	1.308	7.296	0.437	0.103	0.03	0.103	0.03	1.011	0.433	0.201	0.066	0.89985
17	OUMV	-95.026	203.698	2.888	2.558	1.04	7.033	0.456	0.075	0.035	0.075	0.035	0.691	0.297	0.15	0.07	0.88417
18	OUMVA	-94.451	205.143	0.322	2.775	1.368	7.264	0.442	0.067	0.027	0.075	0.028	1.265	0.701	0.054	0.028	0.64390
19	OUMV	-100.933	215.513	3.148	2.37	2.189	6.708	0.323	0.182	0.066	0.182	0.066	2.294	1.214	0.372	0.152	0.61760
20	OUMV	-90.023	193.693	2.02	2.834	1.701	7.251	0.085	0.131	0.032	0.131	0.032	1.612	0.613	0.111	0.037	0.59402
21	OUMV	-98.812	211.271	2.823	2.517	1.113	6.884	0.164	0.064	0.025	0.064	0.025	0.783	0.322	0.113	0.043	0.67928
22	OUMV	-91.985	197.617	3.269	2.426	1.8	6.942	NaN	0.133	0.028	0.133	0.028	1.722	0.669	0.114	0.039	0.72951
23	OUMA	-97.272	208.191	3.736	1.943	1.234	7.648	0.236	0.143	0.032	0.135	0.032	0.795	0.216	0.795	0.216	0.97206
24	OUMV	-94.675	202.997	2.976	2.442	0.797	7.846	0.743	0.053	0.016	0.053	0.016	0.457	0.14	0.142	0.047	0.73617
25	OUMV	-97.882	209.412	2.922	2.609	1.28	7.228	0.404	0.111	0.062	0.111	0.062	0.964	0.584	0.245	0.114	0.67068
		**Means**	**209.376**	**3.025**	**2.394**	**1.538**	**7.241**	**0.334**	**0.131**	**0.049**	**0.13**	**0.049**	**1.358**	**0.642**	**0.313**	**0.117**	

Each parameter, Theta, α, and σ^2, are categorized by non-ornamented (0) and ornamented (1) regimes. Means are model averaged from Akaike Weights.

Supplementary Table 4. Model estimates of AICc selected Generalized Hansen Models from OUwie with root estimated.

Tree	Model	Log Lik	AICc	Theta0	Theta0 s.e.	Theta1	Theta1 s.e.	α0	α0 s.e	α 1	α 1 s.e.	$\sigma^2$0	$\sigma^2$0 s.e.	$\sigma^2$1	$\sigma^2$1 s.e.	Akaike Weights
1	OUMV	-89.22	189.593	2.826	0.899	7.081	0.155	0.159	0.033	0.159	0.033	1.906	0.767	0.144	0.043	0.77767
2	OUMV	-98.707	208.568	2.867	0.856	7.127	0.328	0.091	0.038	0.091	0.038	0.973	0.422	0.155	0.069	0.70501
3	OUMV	-102.96	217.073	2.313	0.975	6.727	0.653	0.058	0.069	0.058	0.069	0.972	1.019	0.144	0.074	0.67629
4	OUMV	-100.345	211.844	2.651	0.911	7.258	0.355	0.071	0.026	0.071	0.026	0.907	0.415	0.112	0.042	0.40957
5	OUMV	-97.41	205.974	1.331	0.993	7.123	0.193	0.18	0.06	0.18	0.06	2.892	1.37	0.177	0.072	0.99940
6	OUMV	-99.073	209.3	2.492	0.919	7.412	0.427	0.087	0.027	0.087	0.027	1.048	0.422	0.159	0.054	0.72593
7	OUMV	-94.41	199.974	2.766	0.852	7.411	0.325	0.111	0.039	0.111	0.039	1.104	0.526	0.152	0.054	0.99893
8	OUMV	-89.446	190.045	1.974	0.891	7.302	0.218	0.112	0.025	0.112	0.025	1.18	0.433	0.114	0.037	0.99997
9	OUMV	-102.953	217.06	2.604	0.801	7.351	0.746	0.067	0.045	0.067	0.045	0.703	0.388	0.176	0.086	0.67274
10	OUMV	-94.444	200.042	2.51	0.796	6.949	0.384	0.103	0.037	0.103	0.037	0.974	0.426	0.174	0.063	0.66349
11	OUMV	-99.088	209.331	2.117	0.85	7.056	0.259	0.168	0.065	0.168	0.065	2.058	0.987	0.267	0.122	0.98990
12	OUMV	-103.287	217.728	3.109	0.859	6.922	0.346	0.155	0.085	0.155	0.085	1.855	0.954	0.315	0.191	0.69572
13	OUMV	-96.893	204.941	2.722	0.806	7.383	0.31	0.099	0.032	0.099	0.032	0.88	0.339	0.172	0.065	0.73303
14	OUMV	-93.182	197.519	2.617	0.832	7.734	0.176	0.163	0.031	0.163	0.031	1.87	0.684	0.159	0.049	0.74596
15	OUMV	-97.013	205.18	2.357	0.94	6.986	0.339	0.141	0.067	0.141	0.067	1.735	0.958	0.191	0.094	0.91977
16	OUMV	-95.285	201.723	2.287	0.876	7.356	0.413	0.101	0.027	0.101	0.027	1.026	0.428	0.18	0.055	0.75431
17	OUMV	-95.525	202.203	2.69	0.817	6.914	0.408	0.083	0.029	0.083	0.029	0.811	0.324	0.151	0.061	0.97920
18	OUMV	-96.548	204.25	2.098	0.812	7.472	0.38	0.11	0.054	0.11	0.054	1.015	0.538	0.18	0.094	0.99190
19	OUMV	-101.095	213.343	2.868	0.98	6.686	0.323	0.169	0.066	0.169	0.066	2.323	1.244	0.33	0.14	0.54485
20	OUMV	-89.866	190.886	2.255	0.922	7.234	0.125	0.132	0.03	0.132	0.03	1.668	0.616	0.107	0.034	0.63948
21	OUMV	-98.902	208.958	2.58	0.937	6.759	0.357	0.072	0.025	0.072	0.025	0.941	0.384	0.108	0.04	0.71095
22	OUMV	-92.011	195.176	2.865	1.038	6.961	0.152	0.131	0.03	0.131	0.03	1.72	0.69	0.112	0.038	0.73712
23	OUMA	-100.18	211.513	2.761	0.704	8.067	0.333	0.104	0.029	0.096	0.028	0.659	0.186	0.659	0.186	0.62368
24	OUMV	-95.664	202.481	2.316	0.676	7.548	0.798	0.047	0.022	0.047	0.022	0.434	0.174	0.14	0.048	0.61723
25	OUMV	-97.823	206.799	2.588	0.824	7.298	0.409	0.096	0.053	0.096	0.053	0.919	0.531	0.204	0.092	0.74029
		**Means**	**204.323**	**2.467**	**0.872**	**7.204**	**0.348**	**0.115**	**0.042**	**0.115**	**0.042**	**1.334**	**0.622**	**0.188**	**0.075**	

Each parameter, Theta, α, and σ^2, are categorized by non-ornamented (0) and ornamented (1) regimes. Means are model averaged from Akaike Weights.

Supplementary Table 5. Table of primary data used in this study

Taxon	Femoral Length (mm) or Alternative Measure	Body Mass (kg)	Cranial Ornament	Time Interval
Abelisaurus comahuensis	Skull length from Sampson and Witmer [1] fig. 2, extrapolated body mass based on *Carnotaurus* skull length, 596 mm [2]	3540	1	35
Acrocanthosaurus atokensis	1180	5250	0	1
Afrovenator abakensis	761	986	0	2
Ajancingenia yanshini	240	24	0	36
Albertosaurus sarcophagus	1020	2934	1	27
Alioramus remotus	560	369	1	23
Allosaurus fragilis	1001	2396	1	3
Anchiornis huxleyi	43.2	0.096	0	46
Anzu wyliei	495	247.8	1	28
Appalachiosaurus montgomeriensis	786	1099.1	1	35
Archeopteryx lithographica	61	0.291	0	40
Aucasaurus garridoi	725 from [2]	847	1	52
Austroraptor cabazai	560 [3]	369	0	36
Avimimus portentosus	188	10.9	0	35
Banji long	123	2.8	0	36
Baryonyx walkeri	Body mass taken from [4]	1981	1	54
Beipiaosaurus inexpectus	250	27	0	31
Buitreraptor gonzalezorum	145	4.741	0	19
Byronosaurus jaffei	Makovicky et al., [5] dentary 85.7 mm, Body mass extrapolated from *Zanabazar* [6] dentary 117 mm	3.102	0	35
Carcharodontosaurus saharicus	1260	5028	0	18
Carnotaurus sastrei	1018	2529.1	1	10
Caudipteryx zoui	149	5.2	0	31
Ceratosaurus nasicornis	759	982	1	8
Citipati osmolskae	397	121.7	0	35
Coelophysis bauri	245	26	0	7

Compsognathus longipes	109.7	0.5	0	21
Concavenator corcovatus	560	368.7	0	45
Conchoraptor gracilis	183	10	0	36
Confuciusornis sanctus	42	0.088	0	24
Cryolophosaurus ellioti	769	1024	1	11
Daspletosaurus torosus	1020	2388	1	27
Deinonychus antirrhopus	440	169.514	0	14
Dilong paradoxus	181	10	0	24
Dilophosaurus wetherilli	552	362	1	6
Dubreuillosaurus valesdunensis	450	182	0	15
Eocarcharia dinops	Sereno and Brusatte [7], frontal 102 mm, Body mass extrapolated from *Acrocanthosaurus* frontal 191 mm [8]	2804	1	14
Eoraptor lunensis	154	5.757	0	4
Eosinopteryx brevipenna	48.5	0.186	0	46
Eotyrannus lengi	Estimated tibia length 535 mm	250	0	25
Epidexipteryx hui	51	0.164	0	38
Erlikosaurus andrewsi	513	278	0	20
Eustreptospondylus oxoniensis	510	253	0	13
Falcarius utahensis	403	128	0	34
Gallimimus bullatus	673	667	0	9
Garudimimus brevipes	371	98	0	20
Giganotosaurus carolinii	1350	6280	1	19
Gobivenator mongoliensis	192 [9]	11.7	0	35
Gorgosaurus libratus	967	2709	1	27
Guanlong wucaii	420	163	1	16
Haplocheirus sollers	214.32	16.7	0	33
Harpymimus okladnikovi	406	131	0	30
Herrerasaurus ischigualastensis	482	227.393	0	5
Hexing qingyi	135	3.766	0	42
Huaxiagnathus orientalis	170	7.96	0	24
Incisivosaurus gauthieri	174	8.5	0	31
Irritator challengeri	Skull roof length [10], body mass extrapolated from	1464	1	53

	Baryonyx [11]			
Jinfengopteryx elegans	70	0.454	0	37
Juravenator starki	53.03	0.186	0	44
Khaan mckennai	194	12.1	0	35
Limusaurus inextricabilis	208 [12]	15.1	0	16
Linheraptor exquisitus	230	20.96	0	10
Lythronax argestes	Loewen et al. [13] tibia length 810 mm, femur estimated 861.7 mm from *Daspletosaurus* MOR 590 as reported in Currie [14], femur 865 mm, tibia 813.1	1478	1	27
Majungasaurus crenatissimus	568	386	1	9
Mapusaurus roseae	1300	5561	1	20
Masiakasaurus knopfleri	202.5	14	0	9
Mei long	81	0.726	0	37
Microraptor zhaoianus	100	1.432	0	41
Monolophosaurus jiangi	From [4]	391.05	1	12
Mononykus olecranus	138.6	4.1	0	9
Nemegtomaia barsboldi	360	88.8	1	9
Neovenator salerii	780	866	0	17
Nqwebasaurus thwazi	52	0.174	0	48
Ornitholestes hermanni	220	18.167	0	22
Ornithomimus velox	435	163	0	27
Oviraptor philoceratops	303	51	1	35
Pelecanimimus polydon	191	12	0	29
Piatnitzkysaurus floresi	548	352	1	12
Proceratosaurus bradleyi	Rahut et al. [15] skull length 251.4 mm, Body mass extrapolated from *Guanlong* [16]	146.3	1	15
Rajasaurus narmadensis	Body mass extrapolate from distal femur breadth compared to *Carnotaurus* [17]	1916	1	23
Raptorex kriegsteini	338	72	0	26
Rinchenia mongoliensis	339	73.2	1	9
Rugops primus	Sereno et al. [18] skull length 315 mm, Body mass extrapolated from *Carnotaurus* skull length 596 mm [2]	1336	1	19
Sapeornis chaoyangensis	81	0.726	0	39

Sauroniops pachytholus	Cau et al. [19], frontal 177.9 mm, Body mass extrapolated from *Acrocanthosaurus* frontal 191 mm [8]	4890	1	19
Saurornithoides mongoliensis	200	13.363	0	36
Saurornitholestes langstoni	225	19.531	0	35
Shenzhousaurus orientalis	191	12	0	31
Shuvuuia deserti	108	1.8	0	35
Sinornithosaurus millenii	148	5.065	0	24
Sinosauropteryx prima	53	0.185	0	24
Sinosaurus triassicus	587	429	1	55
Sinovenator changii	118	2.441	0	37
Sinraptor dongi	869.5	1559	1	16
Sinraptor hepingensis	995	2237	1	16
Skorpiovenator bustingorryi	Juarez Valieri et al. [2] skull length 281 mm. Body mass extrapolated from *Carnotaurus* skull length 596 mm [2]	1192	1	51
Struthiomimus altus	512	276	0	27
Suchomimus tenerensis	1080	3014	0	14
Syntarsus kyantakatae	272	35.98	1	6
Tanycolagreus topwilsoni	355	84.89	0	49
Tarbosaurus bataar	1120	4297	1	23
Teratophoneus curriei	757	974	1	27
Tianyuraptor ostromi	207 [20]	14.9	0	31
Troodon formosus	300	49.35	0	10
Tsaagan mangas	Norell et al. [21] skull length 193.3 mm, Body mass extrapolated from *Linheraptor* [22] skull length 225 mm	18.01	0	35
Tyrannosaurus rex	1350	6168	1	28
Velociraptor mongoliensis	238	23.406	0	10
Xioatingia zhengi	84	0.812	0	47
Xiongguanlong baimoensis	510	401	0	14
Yangchuano magnus	1200	4297	1	16
Yutyrannus huali	850 [23]	1415	1	31
Zanabazar junior	140	4.235	0	23
Zuolong salleei	336	71.1	0	16

Supplementary Table 6. Time interval data used in paleotree time scaling

Time Interval	Start Time (Ma)	End Time (Ma)
1	125	113
2	174.1	145
3	150	145
4	237	228
5	237	208.5
6	199.3	182.7
7	228	210.3
8	161.2	145
9	70.6	65.5
10	83.5	72.1
11	199.3	182.7
12	166.1	163.5
13	164.7	161.2
14	125	100.5
15	167.7	164.7
16	163.5	157.3
17	129.4	125
18	113	93.5
19	99.6	93.5
20	99.6	89.8
21	152	145
22	157.3	145
23	72.1	66
24	130	122.5
25	136.4	124.5
26	129.4	122.5
27	76.5	75.5
28	67	65.5
29	133	128
30	136	125
31	125	125
32	99.6	89.3
33	161.2	158.7
34	124.5	124.5
35	83.5	70.6
36	83.5	65.5
37	125	122.5
38	166.1	157.3
39	125	122.5
40	152.1	145
41	120	111
42	140.2	125.5
43	76.5	75.5
44	155.7	150.8

45	130	125.5
46	167.7	150.8
47	161.2	152
48	145	132.9
49	157.3	152.1
50	80.6	79.9

Supplementary Discussion

Alternative results interpretation. The interpretation of the results supported in this study is that once a lineage gains cranial ornaments then body mass evolution changes trajectories from a more Brownian motion random walk model to that of Brownian motion with a trend. However, it is conceivable that one could interpret the presence of many ornamented species with large body masses in our original data set as that theropod lineages naturally evolve toward larger body sizes and ornamentation is inevitable when a lineage obtains large size. Testing these philosophical differences in a Bayesian framework is possible, yet requires analyses outside the scope of this paper.

Biases of AIC-based OU model inference. Information theoretic approaches to model selection within phylogenetic comparative studies are the most common method to assess results from a suite of analyses. However, critical review of methods such as Akaike Information Criterion (AIC) has revealed some biased tendencies in model selection behavior that has important implications for interpretation within an evolutionary context. Specifically, AIC and AICc were shown to choose overly complex models more often than simpler models, even when the correct model was the simpler version[24,25]. The models that we tested within our OU framework ranged from simple to complex, and as such they possibly suffer from the issues raised of overly complex models being chosen preferentially. Within the current framework of OUwie[26] there is no other option for model assessment; therefore, we provide the Akaike Weights for each model given a specific phylogenetic tree so that the relative confidence in each model can be assessed (Supplementary Tables 3 & 4) in addition to the full estimated model parameters for each evolutionary model available in OUwie recorded in the Supplementary Data 1. As can be seen in the results for models where the optima migrates away from an ancestral state (root.station not estimated, Supplementary Table 3), the OUMV model possesses the majority of weight in virtually all cases, whereas the similarly complex OUMA model yields little weight. Though this does not negate the concerns of prior studies on AICc selection bias, it provides some support that our results may reflect a real biological pattern and not simply model assessment bias.

Supplementary Methods

Phylogenetic Tree. We grafted a phylogenetic tree based on topologies published on various clades including basal theropods and tetanurans[12,19,27–30], tyrannosauroids[13,23,31], ornithomimosaurs[32], and all other coelurosaurs[3,9,33–35]. The femoral/body mass regression of Christiansen and Farina[36] was used to estimate body mass. If femora were not present on a particular taxon their body mass was estimated by scaling another element present in the specimen with the same element and body mass of their closest taxon on the phylogeny. Time Interval

references the time intervals that are required to run the paleotree R package[37]. See Supplementary Table 6 for complete list of intervals.

Sauroniops [19] and *Eocarcharia* [7] each scaled a frontal (177.9 mm and 102 mm, respectively) to that of *Acrocanthosaurus* (NCSM 14345, 191 mm) [8]. We scaled the total skull length of *Proceratosaurus* (251.4 mm) [15] to that of *Guanlong* (280.1 mm) [16]. Likewise, the total skull length of *Tsaagan* (193.3 mm) [21] was scaled to the total skull length of *Linheraptor* (225 mm) [22]. *Byronosaurus* [5] was scaled using only the dentary (85.7 mm) compared to *Zanabazar* (117 mm) [6]. Finally, the body mass of *Lythronax* was extrapolated using comparative tibial lengths between this taxon (810 mm) with that of *Daspletosaurus* (MOR 590, 865 mm) as reported by Currie [14].

Supplementary References

1. Sampson, S. D. & Witmer, L. M. Craniofacial anatomy of Majungasaurus crenatissimus (Theropoda: Abelisauridae) from the Late Cretaceous of Madagascar. *J. Vertebr. Paleontol.* **27,** 32–104 (2007).
2. Juárez Valieri, R. D. & Porfiri, J. D. New information on Ekrixinatosaurus novasi Calvo et al 2004, a giant and massively-constructed Abelisauroid from the '"Middle Cretaceous"'of Patagonia. *Paleontol. Dinosaur. En América Lat.* 161–169 (2010).
3. Novas, F. E., Pol, D., Canale, J. I., Porfiri, J. D. & Calvo, J. O. A bizarre Cretaceous theropod dinosaur from Patagonia and the evolution of Gondwanan dromaeosaurids. *Proc. R. Soc. B Biol. Sci.* **276,** 1101–1107 (2009).
4. Therrien, F. & Henderson, D. M. My theropod is bigger than yours… or not: estimating body size from skull length in theropods. *J. Vertebr. Paleontol.* **27,** 108–115 (2007).
5. Makovicky, P. J., Norell, M. A., Clark, J. M. & Rowe, T. Osteology and relationships of Byronosaurus jaffei (Theropoda: Troodontidae). *Am. Mus. Novit.* 1–32 (2003).
6. Norell, M. A. *et al.* A review of the Mongolian Cretaceous dinosaur Saurornithoides (Troodontidae: Theropoda). *Am. Mus. Novit.* 1–63 (2009).
7. Brusatte, S. L. & Sereno, P. C. Basal abelisaurid and carcharodontosaurid theropods from the Lower Cretaceous Elrhaz Formation of Niger. *Acta Palaeontol. Pol.* **53,** 15–46 (2008).
8. Eddy, D. R. & Clarke, J. A. New information on the cranial anatomy of Acrocanthosaurus atokensis and its implications for the phylogeny of Allosauroidea (Dinosauria: Theropoda). *PLOS One* **6,** e17932 (2011).
9. Tsuihiji, T. *et al.* An exquisitely preserved troodontid theropod with new information on the palatal structure from the Upper Cretaceous of Mongolia. *Naturwissenschaften* 1–12 (2014).
10. Sues, H.-D., Frey, E., Martill, D. M. & Scott, D. M. Irritator challengeri, a spinosaurid (Dinosauria: Theropoda) from the Lower Cretaceous of Brazil. *J. Vertebr. Paleontol.* **22,** 535–547 (2002).
11. Charig, A. J. & Milner, A. C. Baryonyx walkeri, a fish-eating dinosaur from the Wealden of Surrey. *Bull.-Nat. Hist. Mus. Geol. Ser.* **53,** 11–70 (1997).
12. Xu, X. *et al.* A Jurassic ceratosaur from China helps clarify avian digital homologies. *Nature* **459,** 940–944 (2009).
13. Loewen, M. A., Irmis, R. B., Sertich, J. J., Currie, P. J. & Sampson, S. D. Tyrant dinosaur evolution tracks the rise and fall of Late Cretaceous oceans. *PloS One* **8,** e79420 (2013).
14. Currie, P. J. Possible evidence of gregarious behavior in tyrannosaurids. *Gaia* **15,** 271–277 (1998).

15. Rauhut, O. W., Milner, A. C. & Moore-Fay, S. Cranial osteology and phylogenetic position of the theropod dinosaur Proceratosaurus bradleyi (Woodward, 1910) from the Middle Jurassic of England. *Zool. J. Linn. Soc.* **158,** 155–195 (2010).
16. Xu, X. *et al.* A basal tyrannosauroid dinosaur from the Late Jurassic of China. *Nature* **439,** 715–718 (2006).
17. Wilson, J. A. *et al. A new abelisaurid (Dinosauria, Theropoda) from the Lameta Formation (Cretaceous, Maastrichtian) of India*. (Museum of Paleontology, the University of Michigan, 2003).
18. Sereno, P. C., Wilson, J. A. & Conrad, J. L. New dinosaurs link southern landmasses in the mid-Cretaceous. *Proc. R. Soc. Lond. Ser. B* **271,** 1325–1330 (2004).
19. Cau, A., Dalla Vecchia, F. M. & Fabbri, M. A thick-skulled theropod (Dinosauria, Saurischia) from the Upper Cretaceous of Morocco with implications for carcharodontosaurid cranial evolution. *Cretac. Res.* (2012).
20. Zheng, X., Xu, X., You, H., Zhao, Q. & Dong, Z. A short-armed dromaeosaurid from the Jehol Group of China with implications for early dromaeosaurid evolution. *Proc. R. Soc. B Biol. Sci.* **277,** 211–217 (2010).
21. Norell, M. A. *et al.* A new dromaeosaurid theropod from Ukhaa Tolgod (Omnogov, Mongolia). *Am. Mus. Novit.* **3545,** 51 (2006).
22. Xu, X. *et al.* A new dromaeosaurid (Dinosauria: Theropoda) from the Upper Cretaceous Wulansuhai Formation of Inner Mongolia, China. *Zootaxa* **2403,** 1–9 (2010).
23. Xu, X. *et al.* A gigantic feathered dinosaur from the Lower Cretaceous of China. *Nature* **484,** 92–95 (2012).
24. Alfaro, M. E. & Huelsenbeck, J. P. Comparative performance of Bayesian and AIC-based measures of phylogenetic model uncertainty. *Syst. Biol.* **55,** 89–96 (2006).
25. Boettiger, C., Coop, G. & Ralph, P. Is your phylogeny informative? Measuring the power of comparative methods. *Evolution* **66,** 2240–2251 (2012).
26. Beaulieu, J. M. & O'Meara, B. OUwie: analysis of evolutionary rates in an OU framework. *R Package Version* **1,** (2012).
27. Carrano, M. T. & Sampson, S. D. The phylogeny of Ceratosauria (Dinosauria: Theropoda). *J. Syst. Palaeontol.* **6,** 183–236 (2008).
28. Carrano, M. T., Benson, R. B. & Sampson, S. D. The phylogeny of Tetanurae (Dinosauria: Theropoda). *J. Syst. Palaeontol.* **10,** 211–300 (2012).
29. Canale, J. I., Scanferla, C. A., Agnolin, F. L. & Novas, F. E. New carnivorous dinosaur from the Late Cretaceous of NW Patagonia and the evolution of abelisaurid theropods. *Naturwissenschaften* **96,** 409–414 (2009).
30. Smith, N. D., Makovicky, P. J., Hammer, W. R. & Currie, P. J. Osteology of Cryolophosaurus ellioti (Dinosauria: Theropoda) from the Early Jurassic of Antarctica and implications for early theropod evolution. *Zool. J. Linn. Soc.* **151,** 377–421 (2007).
31. Brusatte, S. L. & Benson, R. B. The systematics of Late Jurassic tyrannosauroid theropods from Europe and North America. *Acta Palaeontol. Pol.* **58,** 47–54 (2013).
32. Liyong, J., Jun, C. & Godefroit, P. A New Basal Ornithomimosaur (Dinosauria: Theropoda) from the Early Cretaceous Yixian Formation, Northeast China. *Bernissart Dinosaurs Early Cretac. Terr. Ecosyst.* 467 (2012).
33. Choiniere, J. N., Forster, C. A. & de Klerk, W. J. New information on Nqwebasaurus thwazi, a coelurosaurian theropod from the Early Cretaceous Kirkwood Formation in South Africa. *J. Afr. Earth Sci.* **71,** 1–17 (2012).

34. Godefroit, P. *et al.* Reduced plumage and flight ability of a new Jurassic paravian theropod from China. *Nat. Commun.* **4,** 1394 (2013).
35. Zheng, X., Xu, X., You, H., Zhao, Q. & Dong, Z. A short-armed dromaeosaurid from the Jehol Group of China with implications for early dromaeosaurid evolution. *Proc. R. Soc. B Biol. Sci.* **277,** 211–217 (2010).
36. Christiansen, P. & Fariña, R. A. Mass prediction in theropod dinosaurs. *Hist. Biol.* **16,** 85–92 (2004).
37. Bapst, D. W. paleotree: an R package for paleontological and phylogenetic analyses of evolution. *Methods Ecol. Evol.* **3,** 803–807 (2012).
